# Direct observation of oxygen vacancy-driven structural and resistive phase transitions in La_2/3_Sr_1/3_MnO_3_

**DOI:** 10.1038/ncomms14544

**Published:** 2017-02-23

**Authors:** Lide Yao, Sampo Inkinen, Sebastiaan van Dijken

**Affiliations:** 1 NanoSpin, Department of Applied Physics, Aalto University School of Science, P.O. Box 15100, Espoo, FI-00076 Aalto, Finland

## Abstract

Resistive switching in transition metal oxides involves intricate physical and chemical behaviours with potential for non-volatile memory and memristive devices. Although oxygen vacancy migration is known to play a crucial role in resistive switching of oxides, an in-depth understanding of oxygen vacancy-driven effects requires direct imaging of atomic-scale dynamic processes and their real-time impact on resistance changes. Here we use *in situ* transmission electron microscopy to demonstrate reversible switching between three resistance states in epitaxial La_2/3_Sr_1/3_MnO_3_ films. Simultaneous high-resolution imaging and resistance probing indicate that the switching events are caused by the formation of uniform structural phases. Reversible horizontal migration of oxygen vacancies within the manganite film, driven by combined effects of Joule heating and bias voltage, predominantly triggers the structural and resistive transitions. Our findings open prospects for ionotronic devices based on dynamic control of physical properties in complex oxide nanostructures.

## Abstract (web summary)

An in-depth understanding of oxygen vacancy-driven effects is necessary for the development of functional ionic devices. Using simultaneous high-resolution imaging and resistance probing, Yao *et al*. demonstrate oxygen vacancy-driven structural and resistive transitions between three distinct phases in La_2/3_Sr_1/3_MnO_3_.

Oxygen defects can have profound effects on the properties of transition metal oxides. For instance, electric field-directed migration of oxygen vacancies provides a viable mechanism for the formation, rupture and reconstruction of conducting filaments in insulating oxides^1^,^2^,^3^,^4^,^5^,^6^, an effect used in nanoscale resistive switching devices. In complex oxides where magnetic, ferroelectric and superconducting phases emerge from strong correlations between localized transition metal valence electrons, oxygen vacancies can radically alter a plurality of intrinsic properties via valence changes or structural phase transitions^7^. Examples include pronounced effects of oxygen deficiency on metal-insulator transitions^8^, emergent phenomena at oxide interfaces^9^ and magnetic order^10^. The ability to reversibly control the concentration and profile of oxygen vacancies in oxide nanostructures would open comprehensive prospects for new functional ionic devices. Advancements in this direction require experimental techniques that allow for simultaneous measurements of oxygen vacancy dynamics, atomic-scale structural transitions and macroscopic physical responses.

Here we report on deterministic voltage control of three structural phases with distinctive resistance states in La_2/3_Sr_1/3_MnO_3_ (LSMO) epitaxial films. LSMO of this composition is a popular conductive ferromagnetic perovskite oxide with attractive magnetoresistance and half-metallic properties for spintronics^11^,^12^. Previous studies on single crystals and thin films have shown that transport and magnetic properties of LSMO vary sensitively with oxygen off-stoichiometry^13^,^14^,^15^,^16^. Moreover, at sufficiently large concentrations, ordering of oxygen vacancies in LSMO can cause a transition to the brownmillerite crystal structure^17^,^18^,^19^. Although control over oxygen deficiency can be attained by growth or annealing in various oxygen environments, this approach does not allow material properties to be actively tuned. As a scientific alternative, *in situ* transmission electron microscopy (TEM) has emerged as a powerful technique for the analysis of structural transitions and redox processes following the application of voltage pulses^3^,^4^,^5^,^6^,^20^,^21^,^22^. In this work, we employ simultaneous high-resolution TEM imaging and resistance probing to investigate the dynamics of oxygen-vacancy-induced structural transitions and concurrent changes in electrical transport. Our data demonstrate how reversible migration of oxygen vacancies towards and away from the LSMO contact area results in controlled switching between three uniform structural phases during the application of short voltage pulses. The atomic-scale evolution of new structural phases in LSMO correlates directly with the electrical resistance of the sample.

## Results

### *In situ* TEM experiments

We conducted the experiments using aberration-corrected TEM and a double-tilt probing holder with a piezo-controlled metal tip. The samples, consisting of a 20 nm-thick LSMO film on a conducting Nb-doped SrTiO_3_ (STO) substrate, were grown by pulsed laser deposition and prepared into cross-sectional TEM wedges by mechanical polishing and Ar ion milling. Electrical contacts to the STO side of the sample were made by placing one of the wedges on a half Cu grid. After mounting the TEM sample into the microscope, we brought the electrically grounded tip into contact with the LSMO film (Supplementary Fig. 1). This produced an effective contact area with a diameter of ∼30 nm. The electrical resistance was continuously monitored and scanning TEM (STEM) images with high-angle annular dark field contrast were simultaneously recorded after application of short triangular voltage pulses to the STO side of the specimen. We used the STEM mode deliberately to limit electron beam exposure to a small spot with a diameter of about 1.5 Å at a time. Tests involving repeated imaging after the application of a voltage pulse confirmed that STEM imaging did not affect the resistance state of the sample, thus ruling out any electron beam-induced effects. The Methods section provides more details on the experiments.

### Resistive switching and structural phase transitions

**Figure 1:**
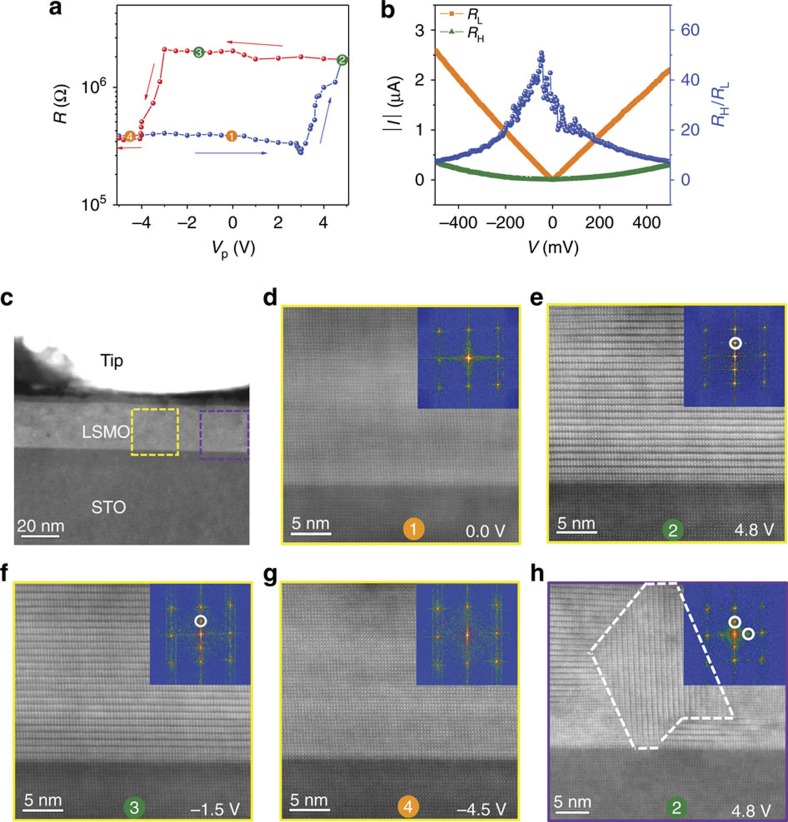
Two-level resistive switching driven by structural phase transitions. (**a**) *In situ* TEM resistive switching curve for a LSMO film on top of a conducting SrTiO_3_ substrate. The resistance is measured at *V*_m_=0.5 V after the applications of triangular voltage pulses with maximum voltage *V*_p_ and a duration of 100 ms. (**b**) *I*–*V* curves for the same film, measured after switching to the low (orange squares) and high (green triangles) resistance states. The blue data points indicate the resistance ratio as a function of bias voltage. (**c**) Cross-sectional STEM image of the contact between the metal tip and the LSMO film. (**d**–**h**) STEM HAADF images and corresponding fast Fourier transform (FFT) patterns of the sample within the contact area, as indicated by the dashed boxes in **c**, at several stages of the bipolar resistive switching process. The images of **d**–**h** and data in **a** were collected simultaneously (coloured dots indicate matching measurements). The STEM images indicate that switching to the high-resistance state by positive voltage pulses coincides with a uniform structural transition to the brownmillerite phase ((1)→(2)), whereas the low-resistance perovskite structure is re-established by negative voltage pulses ((3)→(4)). In some areas (see **h**), the horizontal brownmillerite phase coexists with a vertical brownmillerite structure.

Figure 1 presents *in situ* TEM results on resistive switching in the LSMO film. The resistance data (Fig. 1a) and STEM images (Fig. 1d–h) are measured instantaneously after the application of short (100 ms) triangular voltage pulses between the metal tip and conducting STO substrate. An increase of pulse amplitude (*V*_p_) triggers a transition to a higher resistance state for positive voltages. The resistive switching process directly correlates with a modification of the LSMO lattice structure. In saturation ((2) in Fig. 1a), the original perovskite lattice (Fig. 1d) has completely transformed into an ordered superstructure with a periodicity of two unit cells (Fig. 1e). The new structural phase and the corresponding high-resistance state are stable upon reduction of *V*_p_ until, at large enough negative voltages, the low resistance state and original perovskite structure are re-established ((4) in Fig. 1a,g). The switching process is thus bipolar and non-volatile. Repeated cycling of the voltage pulses gives identical results, signifying good reproducibility of the resistive switching process (Supplementary Fig. 2). The modulated planes of the high-resistance LSMO superstructure are mostly oriented parallel to the substrate, but smaller domains with vertically aligned planes also form to relax epitaxial strain^23^. An example is shown in Fig. 1h. The transport characteristics of the two structural phases in LSMO are different (Fig. 1b). Ohmic behaviour is measured on the perovskite structure, which is anticipated for metallic La_1−*x*_Sr_*x*_MnO_3_ films with *x*=1/3^11^. In contrast, nonlinear *I*–*V* curves are recorded for the electrically induced LSMO superstructure.

**Figure 2:**
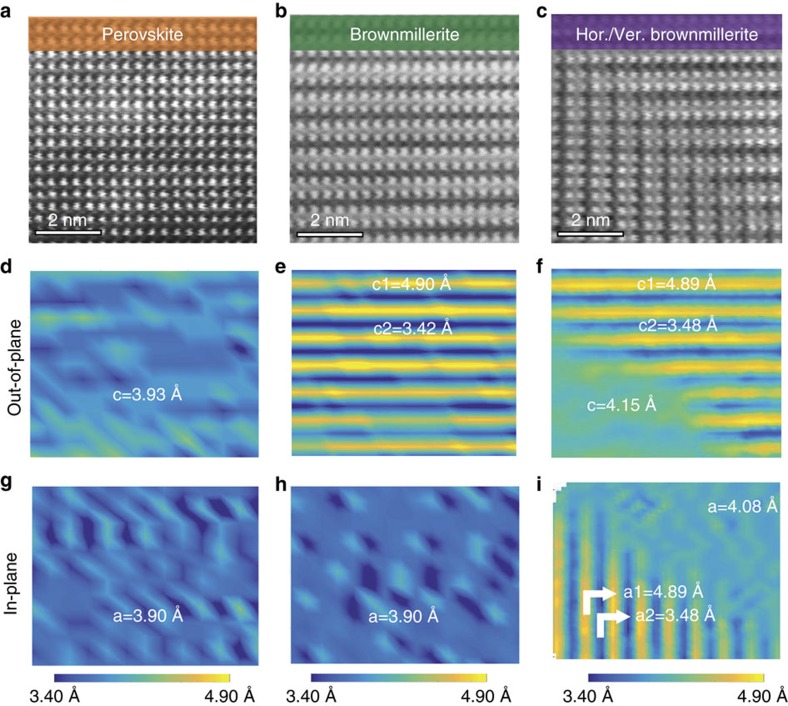
Ordering of oxygen vacancies. STEM images with HAADF contrast of the LSMO film showing the perovskite structure (**a**), the horizontal brownmillerite phase (**b**) and a vertical brownmillerite domain (**c**). The images are recorded along the [100] direction of the substrate. Maps of the out-of-plane and in-plane lattice spacing between La/Sr ions are shown in **d**–**f** and **g**–**i**, respectively.

Figure 2 shows high-resolution STEM images and lattice spacing maps of the different structural phases in LSMO. The as-grown perovskite LSMO film is epitaxial and exhibits full coherency with the STO substrate (*a*=3.90 Å). The same structure is reproducibly obtained after completion of a full switching cycle. The superstructure with a periodicity of two unit cells corresponds to the brownmillerite phase of LSMO^17^,^18^,^19^. Ordering of oxygen vacancies drives the transition into the brownmillerite structure. In the ideal La_2/3_Sr_1/3_MnO_2.5_ brownmillerite lattice, oxygen depletion is confined to every second MnO plane along the modulation direction. This reduces the coordination of Mn cations (MnO_6_ octahedra are replaced by MnO_4_ tetrahedra) and enlarges the spacing between planes with La/Sr ions (*c*_1_ in Fig. 2e). In contrast, unit cells containing oxygen stoichiometric MnO_2_ have a reduced lattice parameter (*c*_2_ in Fig. 2e). Regular modulations of high-angle annular dark field contrast in the STEM images, which are characteristic for oxygen vacancy ordering^18^,^24^, and alterations in lattice spacing are evident for both the horizontal and vertical brownmillerite phases (Fig. 2c). Mixed ionic-electronic conduction in the brownmillerite lattice causes the higher resistance state and nonlinear *I*–*V* transport characteristics (Fig. 1b)^25^.

STEM electron energy-loss spectroscopy (EELS) data confirm the increase of oxygen vacancies in the LSMO film during resistive switching at positive voltage pulses and re-oxidation of LSMO at negative voltages (Supplementary Fig. 3). STEM-EELS spectra also indicate that the oxidation state of STO underneath the LSMO film changes during repeated switching between structural phases. When the concentration of oxygen vacancies is high in LSMO (brownmillerite phase), it is relatively low in STO and vice versa. We note, however, that the changes in the STEM-EELS spectra of STO are relatively small. Vertical migration of oxygen vacancies between the LSMO film and STO substrate is thus limited and its effect on structural and resistive phase transitions in LSMO is weak.

**Figure 3:**
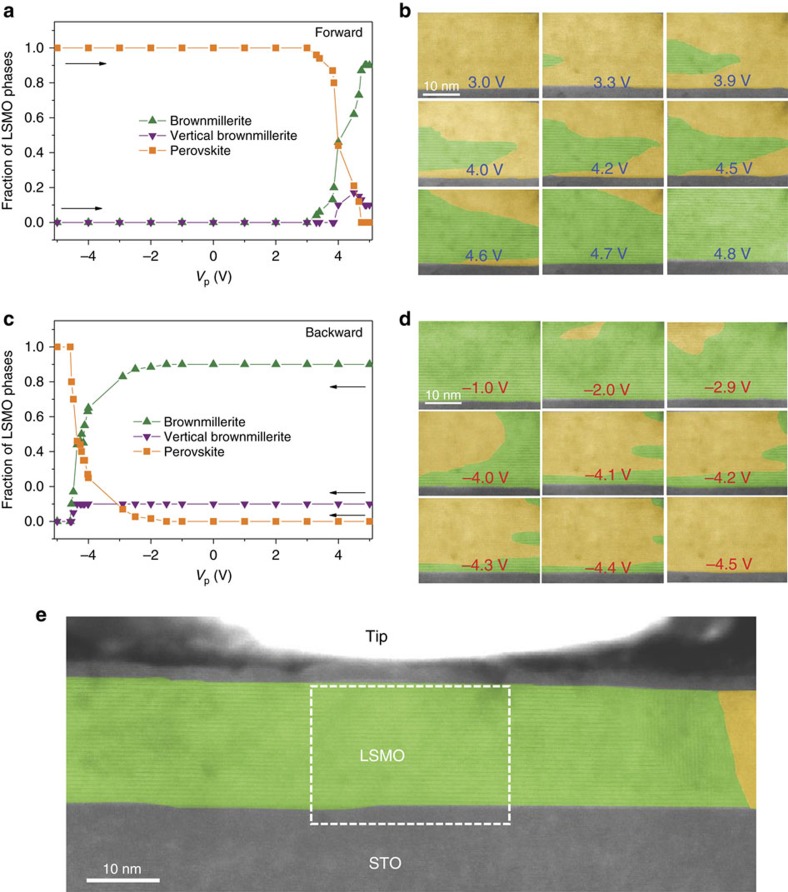
Evolution of structural phases during resistive switching. (**a**,**c**) Areal fraction of various LSMO phases during the resistive switching experiments of Fig. 1. The forward and backward branches of the switching curves are plotted separately for clarity. Data were collected from a 50 nm-wide LSMO area underneath the metal tip after several switching cycles. (**b**,**d**) STEM images illustrating the growth of brownmillerite (green) and perovskite (yellow) structural phases in the forward (**b**) and backward (**d**) branches of the resistive switching curve. The images have been coloured to more clearly visualize the structural transition. (**e**) Lower resolution STEM image of the saturated high-resistance state. The electrically induced brownmillerite structure (green) forms uniformly over a width of about 100 nm. The dashed line indicates the image area of the results shown in **b**,**d**.

Our *in situ* TEM technique allows for direct mapping of structural information onto resistive switching curves. In Fig. 3, this is done for the fraction of various LSMO phases in the sample contact area. In the experiment, the brownmillerite phase nucleates at a voltage pulse of *V*_p_=+3.3 V. A further increase of *V*_p_ enlarges the brownmillerite domains at the expense of the original perovskite lattice. This process, visualized by the STEM images in Fig. 3b, continues until the entire LSMO film in the contact area has transformed into the brownmillerite phase. The resistance starts to increase at the onset of the structural phase transition and it saturates after the brownmillerite structure has spread uniformly. Direct correlations between the structural and resistive phases are also measured for the backward branch of the switching curves (Fig. 3c,d). Lower resolution STEM images of the sample indicate that the brownmillerite phase extends well beyond the contact area in saturation (Fig. 3e). The structure of the LSMO film is changed uniformly by the application of voltage pulses over a length of ∼100 nm.

The STEM images of Fig. 3b,d contain information about the distribution of oxygen vacancies and their migration dynamics. New structural domains nucleate predominantly in the middle of the LSMO film rather than at the STO/LSMO interface and domains grow preferentially along the horizontal direction. Both observations suggest that lateral migration of oxygen vacancies within the LSMO film triggers the switching events. In this picture, oxygen vacancies migrate horizontally from or towards the LSMO contact area when large enough negative or positive voltage pulses are applied to the STO substrate. Vertical migration of oxygen vacancies across the STO/LSMO interface is less evident. If dominant, one would expect domains to nucleate at the interface, which is not the case. Instead, the late dissolution of the brownmillerite phase at the STO/LSMO interface (Fig. 3d) suggests that oxygen vacancy migration from the LSMO film into the STO substrate is limited, in agreement with STEM-EELS analysis (Supplementary Fig. 3). The results of our switching experiments imply a lower activation energy for oxygen vacancy migration in LSMO compared with STO and/or the presence of a migration barrier at the interface^26^. Another effect that could contribute to the elongated shape of structural domains is migration anisotropy within LSMO. Lattice strains in perovskite oxides are known to affect the diffusion coefficient via a change of interatomic bond lengths^27^,^28^, favouring oxygen migration perpendicular to the elongated unit cell^29^. Elongation of the out-of-plane lattice spacing in an oxygen-deficient LSMO film would thus favour horizontal oxygen vacancy migration. Oxygen vacancy migration in brownmillerites is also anisotropic. In this phase, the planes of the superstructure form preferred migration paths^10^. Preferred horizontal growth of the brownmillerite phase is also observed when oxygen vacancies are formed during the first switching event at positive voltages, as illustrated by the STEM images of Supplementary Fig. 4. Analyses of the out-of-plane lattice spacing during the different switching stages further reveal that the lattice parameters of the newly formed structural phases saturate quickly (Supplementary Fig. 5). The oxygen content of brownmillerite domains within a perovskite background (or vice versa) is thus rather homogeneous, and their growth is driven by oxygen vacancy ordering at domain boundaries.

**Figure 4:**
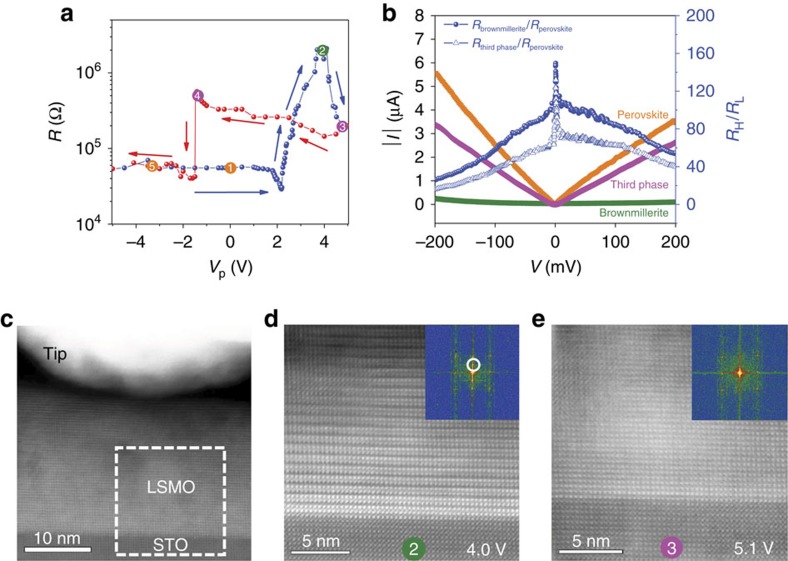
Three-level resistive switching. (**a**) *In situ* TEM resistive switching curve for a LSMO film on top of a conducting SrTiO_3_ substrate. In this experiment, the resistance is measured at *V*_m_=0.2 V after the applications of triangular voltage pulses (*V*_p_) with a duration of 10 ms. Compared with Fig. 1, a third phase with intermediate resistance is formed when the magnitude of positive voltage pulses is further increased after the brownmillerite phase has formed (transition (2)→(3)). The third phase exhibits a homogeneous perovskite structure with an enlarged out-of-plane lattice spacing. At negative bias voltage, a nearly abrupt switch to the original low-resistance perovskite structure occurs. (**b**) *I*–*V* curves for the same film, measured after switching to the low-resistance perovskite phase (orange symbols), the high-resistance brownmillerite phase (green symbols) and the third phase with intermediate resistance (pink symbols). The blue data points indicate the resistance ratios of the two electrically induced structural phases with respect to the as-grown perovskite LSMO film. We note that the differences in resistance between corresponding phases in Figs 1 and 4 are caused by a dissimilar contact resistance between the metal tip and LSMO film in these experiments. (**c**) Cross-sectional STEM image of the contact between the metal tip and LSMO film. (**d**,**e**) STEM HAADF images and corresponding fast Fourier transform (FFT) patterns of the sample within the contact area, as indicated by the dashed box in **c**, just before and after the second structural phase transition ((2)→(3)). More details are provided in the Supplementary Information (Supplementary Figs 6–8).

Resistive switching to a distinctive third state is observed when the voltage pulses are increased further after the high resistance brownmillerite phase has formed. An example is shown in Fig. 4. Here, the orange and green dots again correspond to the perovskite and brownmillerite phases. During the second resistive switching event ((2)→(3)), the characteristic brownmillerite superstructure transforms into a uniform perovskite-like lattice with an enhanced out-of-plane lattice spacing of *c*=4.15 Å (Fig. 4 and Supplementary Figs 6 and 7). This lattice expansion (compared with the original perovskite structure) is caused by randomly distributed oxygen vacancies^19^. For perovskite oxides it is well established that the presence of oxygen vacancies results in an expansion of the crystal lattice due to under-bonding caused by two extra electrons from the missing oxygen located in nonbonding orbitals^30^,^31^. This phenomenon, known as chemical expansion, is most pronounced for perovskites containing transition metal ions that can adopt a variety of valence states such as Mn in LSMO.

STEM-EELS measurements indicate that the third structural phase is more oxygen deficient than the brownmillerite phase (Supplementary Fig. 3). Thus, the oxygen vacancy concentration in the LSMO contact area increases monotonically with the strength of positive voltage pulses. The resistance, on the other hand, first increases when the LSMO film transforms into the brownmillerite structure, but it decreases to an intermediate resistance level after stabilization of the third phase. To explain this non-monotonic variation of resistance with oxygen vacancy concentration, the electronic structures of the different phases need to be considered. In this study, epitaxial LSMO films are used. LSMO of this composition is a good metallic conductor in the original perovskite phase^11^,^12^. Metallic transport in LSMO originates from hopping of *e*_g_ electrons between adjacent Mn^3+^ and Mn^4+^ ions. A reduction of oxygen content changes the Mn^3+^/Mn^4+^ ratio and, thereby, the electrical resistance. Starting from optimally doped LSMO (lowest resistance state), the introduction of oxygen vacancies shifts the effective doping level into the under-doped region of the LSMO phase diagram, in which strongly metallic transport is gradually converted to more insulating behaviour^14^. This effect explains the higher resistance of the third phase compared with the original perovskite structure. In contrast, LSMO with a brownmillerite structure is a mixed ionic-electronic conductor and, hence, its resistance depends on temperature. At room temperature, the resistance of brownmillerites tends to be higher than that of the original perovskite structure^10^. Because of non-metallic transport in the brownmillerite phase, the corresponding *I*–*V* curves are nonlinear (Figs 1b and 4b). Thus, in our experiments we start with an optimally doped metallic LSMO film. The accumulation of oxygen vacancies first changes the resistance by transforming LSMO into a mixed ionic-electronic conductor (high-resistance brownmillerite phase). Hereafter, LSMO is switched back to an electronic conductor, but with considerable under-doping (third phase). The observed variation of resistance with oxygen content is different from many other oxides that are used in resistive switching studies^1^,^2^. In most experiments, the oxides are insulating in the initial state and the introduction of oxygen vacancies lowers the resistance (for example, via filament formation).

The low-resistance perovskite structure is re-established when negative voltage pulses are applied to the third phase ((4)→(5) in Fig. 4a). The intermediate resistance state of the third phase is thus also non-volatile and reversible. Back switching to the perovskite structure is relatively abrupt. Detailed STEM measurements of the (4)→(5) switching event indicate that the structural transformation is completed within a voltage range of only ∼0.2 V (Supplementary Fig. 8). The brownmillerite structure is briefly formed as intermediate phase during this transition.

### Electro-thermal simulations

**Figure 5:**
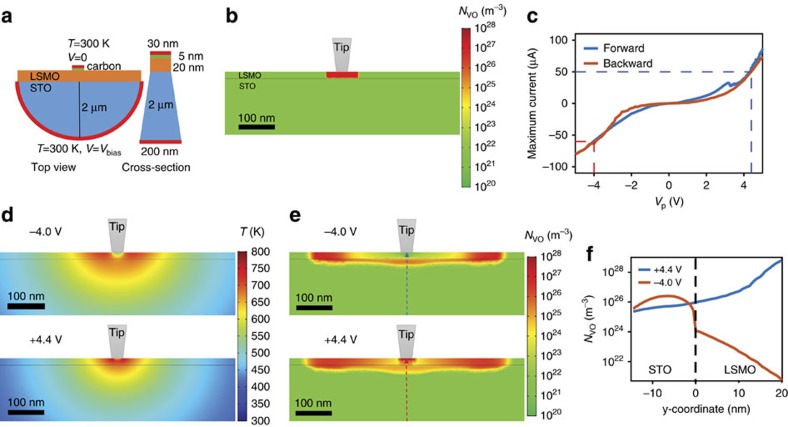
Electro-thermal simulations. (**a**) Schematic of the simulation geometry (not to scale). (**b**) Initial distribution of oxygen vacancies (*N*_VO_) in the LSMO film and SrTiO_3_ (STO) substrate. (**c**) Experimentally measured maximum current during the application of triangular voltage pulses with strength *V*_p_. The data correspond to the *in situ* TEM experiments of Fig. 1. The dashed lines indicate experimental conditions for perovskite-brownmillerite (blue) and brownmillerite-perovskite (red) phase transitions. (**d**) Simulated temperature distribution during the application of negative and positive voltage pulses (*V*_p_=–4.0 V and *V*_p_=+4.4 V). The maximum current during these simulations amounts *I*_max_=–59 μA and *I*_max_=+50 μA, respectively, in agreement with *in situ* TEM experiments (see **c**). (**e**) Distribution of oxygen vacancies after the application of –4.0 V and +4.4 V pulses with a duration of 100 ms. (**f**) Oxygen vacancy concentration profiles underneath the tip in the LSMO film and STO substrate after the application of negative and positive voltage pulses. The profiles in **f** are extracted along the vertical dashed lines in (**e**).

To obtain a more detailed understanding of resistive switching and the dynamics of oxygen vacancies, we performed numerical simulations using an electro-thermal model^32^. In the simulations, a drift-diffusion equation for oxygen vacancies, a continuity equation for electrical conduction and an equation for Joule heating were solved in a self-consistent manner (see Methods for details). To mimic the experimental conditions, we used a metal tip/LSMO film/Nb-doped STO configuration and assumed a uniform region with a length of 100 nm and an oxygen vacancy concentration of *N*_VO_=8 × 10^27^ m^−3^ (Fig. 5a,b). Triangular voltage pulses with a duration of 100 ms were applied to this structure. The strength of the pulses were calibrated with respect to the resistive switching experiments of Fig. 1 (see Fig. 5c and Supplementary Fig. 9). In the experiments, a voltage pulse of *V*_p_=+4.4 V switches the sample into the high-resistance state, while backswitching to the low-resistance configuration is completed at *V*_p_=–4.0 V. Figure 5d shows the simulated temperature distribution during the application of triangular voltage pulses with *V*_p_=–4.0 V and *V*_p_=+4.4 V, respectively. Joule heating temporarily raises the temperature of the LSMO film and STO substrate to >600 K in a semi-circular area with a radius of 100–200 nm around the tip/sample contact. This heating suffices for an electro-thermal redistribution of oxygen vacancies. Figure 5e shows the oxygen vacancy concentration after the application of negative and positive voltage pulses. Under negative bias conditions, positively charged vacancies migrate away from the contact area towards the negative potential on the STO substrate (electric-field maps are provided in Supplementary Fig. 10). Reversal of the pulse polarity produces the opposite outcome. Now, oxygen vacancies migrate back into the contact area of the LSMO film under the combined effect of Joule heating and applied electric field. The line profiles of Fig. 5f indicate a modulation of oxygen vacancy concentration in LSMO by several orders of magnitude after consecutive positive and negative voltage pulses. The simulations of Fig. 5 confirm that the oxygen concentration in the contact area varies mostly by horizontal migration of vacancies within the LSMO film, as suggested by the experimental results of Fig. 3b,d. Vertical migration of oxygen vacancies between the LSMO film and STO substrate is also simulated (in agreement with STEM-EELS data of Supplementary Fig. 3), but the effect is smaller and thus contributes less to the structural and resistive phase transitions in LSMO.

## Discussion

Active and reversible control over oxygen vacancy profiles in well-defined areas of complex oxide heterostructures, as demonstrated here, can be utilized to control material properties in a deterministic and non-volatile manner. Resistive switching in the LSMO/STO system is not based on dielectric breakdown via the formation of conducting filaments in an insulating oxide. Instead, our *in situ* TEM data demonstrate how electro-thermal oxygen vacancy migration triggers the nucleation and growth of uniform structural phases in epitaxial LSMO films. The newly formed phases and corresponding resistance states are non-volatile and stable over time, as demonstrated by *in situ* TEM data in Supplementary Figs 11–13. The distinctive electric resistivity of the structural phases could be used to realize multilevel resistive memories. Beyond resistive switching, the results provide a clear path to active ionic control of other properties of transition metal oxides, including magnetism, ferroelectricity and superconductivity. Transfer of this ionotronics concept to lithographically defined oxide nanostructures is anticipated to promote the emergence of new devices with ionically controlled functions.

## Methods

### Film growth

We epitaxially grew the LSMO films by pulsed laser deposition on conducting single-crystal STO substrates with a Nb doping concentration of 0.7 wt%. Before film growth, the substrates were etched in buffered hydrofluoric acid for 30 s and annealed in oxygen atmosphere at 950 °C for 1 h. This process resulted in TiO_2_-terminated substrate surfaces, as confirmed by atomic force microscopy images showing regular terraces with straight and one unit cell high step edges. The LSMO films were deposited at 700 °C in an oxygen partial pressure of 0.5 mbar. A pulse repetition rate and laser fluence of 4 Hz and 2.5 J cm^−2^ were used. After growth, we cooled the samples down in ∼30 min in 0.5 mbar of oxygen. In all experiments, the thickness of the LSMO film was 20 nm.

### *In situ* TEM experiments

Concurrent microstructural analyses and resistive switching measurements were carried out using a JEOL 2200FS TEM with double Cs correctors and a piezo-controlled *in situ* TEM holder (HE150 electrical probing holder, Nanofactory AB). The following steps were used to fabricate cross-sectional TEM specimens from the LSMO/STO samples. First, we glued Si substrates to each side of the sample. The thus formed Si/sample/Si sandwich was cut into thin slices. Next, a MultiPrep polishing system (Allied High-Tech) polished one of the slices into a wedge. The specimen was subsequently mounted on a half Cu grid and further polished by Ar ion milling. Before mounting the grid onto the *in situ* TEM holder, we unglued the Si from the wedge using acetone. After Si removal, the piezo-controlled metal (Pt/Ir) tip could contact the LSMO film. To make an electrical contact between the conducting STO substrate and the half Cu grid, we used silver paste. From the ratio between the plasmon loss and zero loss peaks in EELS spectra, the thickness of the TEM specimen in the contact area was estimated as 30 nm. Supplementary Fig. 1 shows a schematic of the measurement configuration and a picture of the *in situ* TEM holder.

The *in situ* TEM measurement procedure was as follows. First, we ensured a good electrical contact by monitoring the current between the Pt/Ir metal tip and half Cu grid during a piezo-controlled approach under a bias voltage of 0.2 V. Between experiments, the contact resistance varied, depending on the amount of amorphous carbon between the tip and the LSMO film. After establishing a stable contact, we applied triangular voltage pulses with a fixed duration. After each voltage pulse, the electrical resistance and STEM images were simultaneously measured. The resistance did not change during imaging. Electron-beam-induced changes of the LSMO film structure can therefore be ruled out. This contrasts with previous experiments in which electron-beam exposure of large sample areas in high-resolution TEM mode directly caused structural transitions^33^. In the resistive switching experiments of Figs 1, 3 and 4, we deliberately used STEM to limit electron-beam exposure to a very small area (spot diameter of ∼1.5 Å) at a time. The magnitude of the voltage pulse was increased in a step-like manner and swept between negative and positive values for several cycles. High-resolution STEM images (Fig. 2) and EELS spectra were separately recorded at zero bias voltage after distinct structural phases were formed by appropriate voltage pulses. We used Matlab software and a script for two-dimensional Gaussian fits to extract lattice spacing maps from the STEM images. For the analysis of EELS core-loss peaks, background subtraction was performed using a power-law fit. The Pearson method^34^ was used for quantitative analysis of the Mn white line intensity, providing a direct comparison of the Mn valence state for the different structural phases of LSMO.

### Electro-thermal simulations

Finite element analysis of electric current, Joule heating and migration of oxygen vacancies in the *in situ* TEM sample was performed in COMSOL Multiphysics 5.2. We adapted the numerical modelling approach from previous work on resistive switching in oxides with conductive filaments^32^. The simulation geometry consisted of a semicircular STO substrate with a radius of 2 μm and a metallic contact at the back. The semicircle approximates the shape of equipotential surfaces found in large-scale simulations and the selected radius was sufficiently large so that a temperature boundary condition of 300 K on the back edge did not affect the results. The thickness of the LSMO film was 20 nm. The metallic tip contacting the TEM specimen had a diameter of 30 nm and a 5 nm-thick conducting layer (representing the amorphous carbon layer in the experiments) separated the tip from the LSMO film. The thickness of the cross-sectional TEM specimen was set to 30 nm for the LSMO film and we used a wedge shape (30–200 nm) for the STO substrate. Figure 5a shows a schematic of the simulation geometry. To closely mimic the experiments, we assumed an initial state with uniformly distributed oxygen vacancies in a 100 nm-wide LSMO area underneath the tip (Fig. 5b). The concentration of oxygen vacancies in this area was set to *N*_VO_=8 × 10^27^ m^−3^. This density corresponds to that of the ideal LSMO brownmillerite lattice. In the remainder of the LSMO film and STO substrate, *N*_VO_ was initially set to 10^22^ m^−3^. Triangular voltage pulses with a duration of 100 ms were applied to the backside of the STO substrate (the metal tip was grounded). To account for the total resistance of the experimental setup, a contact resistance of 10^−8^ Ωm^2^ was applied on the outer edge of the simulation geometry. This method enabled us to simultaneously calibrate the strength of the triangular voltage pulses and the maximum electric current to experimental conditions (Supplementary Fig. 9). Joule heating in the electro-thermal simulations and *in situ* TEM experiments are thus comparable.

The electric potential and current were calculated using the current continuity equation


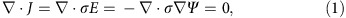


where *J* is the current density, *σ* is the conductivity, *E* is the electric field and *Ψ* is the electric potential. For the temperature dependence of *σ* in the LSMO film and the Nb-doped STO substrate, we used data from refs 35, 36. The room temperature conductivities of LSMO and Nb-doped STO were set to 67 and 20 Scm^−1^, respectively. Variation of the two conductivities with the concentration of oxygen vacancies was taken into account. In LSMO, the conductivity was assumed to decrease linearly by a factor 10 for *N*_VO_ ranging from 10^22^ m^−3^ to 8 × 10^27^ m^−3^. In the same range, the conductivity of STO increased by a factor 10. The temperature (*T*) of the TEM specimen was calculated using the heat equation


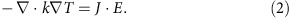


Here, the thermal conductivity (*k*) was set to 2 W/mK for LSMO^12^ and it was allowed to vary with temperature for STO^36^. As the time scale of the experiment is much larger than the time scale of transient effects in temperature and electric current, we solved equations 1 and 2 using these steady-state formulations. Finally, the time-dependent evolution of the oxygen vacancy concentration (*N*_VO_) was calculated using the drift-diffusion equation


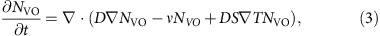


where *D* is the diffusion coefficient (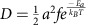), *v* is the drift velocity (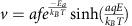) and *S* is the Soret coefficient for thermal diffusion (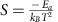). The other parameters are the distance between oxygen sites in the perovskite lattice *a*, attempt-to-escape frequency *f* (10^12^ Hz), activation energy for oxygen vacancy migration *E*_a_ (0.7 eV for LSMO^37^ and 1.1 eV for STO^38^), and the electric charge of oxygen vacancies *q* (+2e). We note that good correspondence between the electro-thermal simulations and STEM-EELS data (Supplementary Fig. 3) is obtained only when a large activation energy for oxygen vacancy migration in STO is assumed. A reduction of this parameter leads to a simultaneous increase (decrease) of oxygen vacancy concentration in LSMO and STO (near the STO/LSMO interface) for positive (negative) voltage pulses, in contrast with STEM-EELS spectra.

### Data availability

The data that support the findings of this study are available from the corresponding authors upon request.

## Additional information

**How to cite this article:** Yao, L. *et al*. Direct observation of oxygen vacancy-driven structural and resistive phase transitions in La_2/3_Sr_1/3_MnO_3_. *Nat. Commun.* **8,** 14544 doi: 10.1038/ncomms14544 (2017).

**Publisher's note**: Springer Nature remains neutral with regard to jurisdictional claims in published maps and institutional affiliations.

## Supplementary Material

Supplementary Information Supplementary Figures, Supplementary Notes and Supplementary References
