# Supplementary material for: Direct observation of oxygen vacancy-driven structural and resistive phase transitions in La2/3Sr1/3MnO3
Source: Nat Commun. 2017 Feb 23;8:14544. doi: 10.1038/ncomms14544 (PMC5331213; doi:10.1038/ncomms14544)
Supplement: Supplementary Information — Supplementary Figures, Supplementary Notes and Supplementary References [file ncomms14544-s1.pdf]

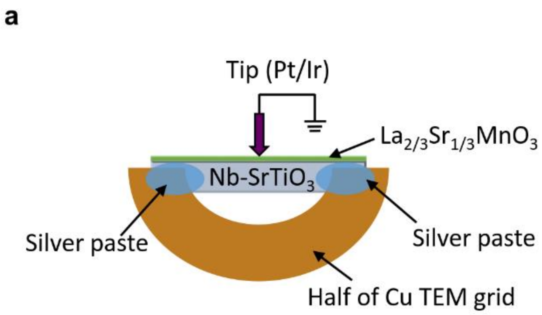

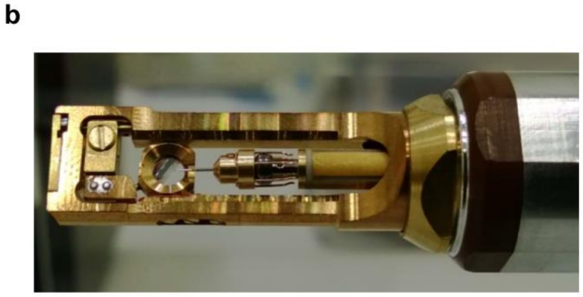

Supplementary Figure 1. a, Schematic illustration of the *in situ* TEM measurement geometry. The wedge-like LSMO/STO specimen is mounted on a half Cu TEM grid. The LSMO side of the sample is contacted by a piezo-controlled metal tip. Silver paste is used to make electrical contacts between the conducting STO substrate and the Cu grid. During the switching experiments, the tip is grounded, and triangular voltage pulses are applied to the TEM grid. **b**, Picture of the *in situ* TEM probing holder.

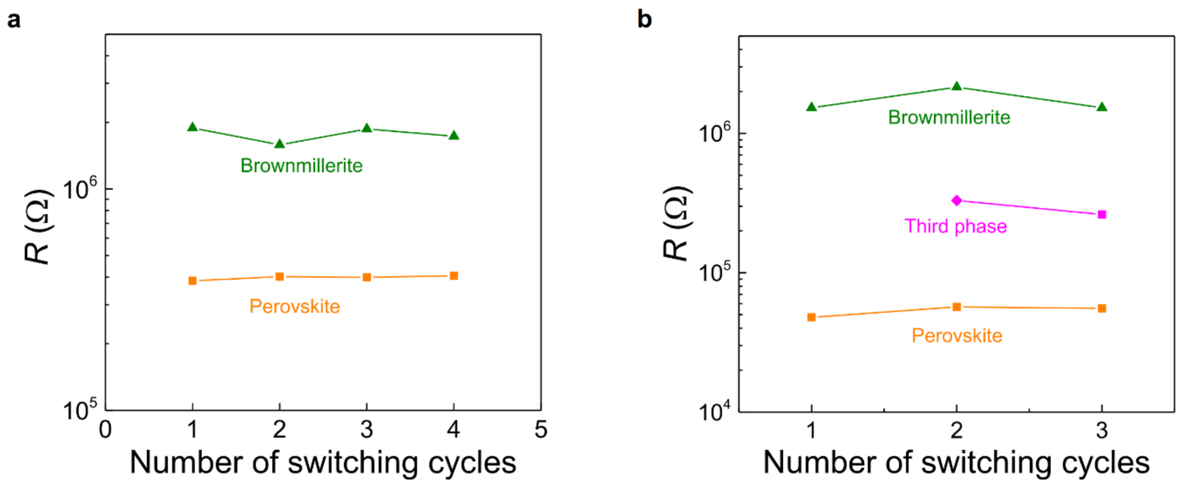

Supplementary Figure 2. a,**b**, Resistance of different structural phases during subsequent voltage-pulse cycles. The measurements in (**a**) and (**b**) correspond to the experiments in Figures 1 and 4 of the main manuscript. Both two-level and three-level resistance switching effects are reproducible.

Supplementary Note 1. STEM-EELS analysis

The initial perovskite structure, brownmillerite lattice, and third phase of the LSMO film were characterized using STEM-EELS. The O K edge fine structure in EELS spectra provides information on excitations from O 1s electrons to 2p bands. The pre-peak of the LSMO O K edge correlates with the occupation of the Mn 3d band, providing information on the oxidation state of Mn ions. A drop in the pre-peak intensity and a shift towards higher energy losses indicate an increase in oxygen vacancy concentration [1-3]. Supplementary Figure 3a depicts STEM-EELS spectra for the different structural phases of LSMO. The pre-peak of the O K edge is reduced for the brownmillerite structure (both horizontal and vertical) in comparison to the EELS spectrum of the original perovskite lattice (yellow curve). This observation confirms a reduction of the Mn oxidation state via electro-thermal migration of oxygen vacancies towards the LSMO contact area during the application of positive voltage pulses. After the perovskite structure is re-established by negative voltage pulses (blue curve), the oxidation state of Mn ions is also restored. The pre-peak of the O K edge is further reduced for the perovskite structure

with enhanced out-of-plane lattice spacing (third phase, pink curve). The concentration of oxygen vacancies in the third phase is thus higher than that of the oxygen-deficient brownmillerite phase. The conclusions on oxygen vacancy concentration are further supported by a comparison of the Mn white line L_3/L_2 peak ratio (Supplementary Figure 3b). Since an increase of the L_3/L_2 peak ratio implies a lowering of the Mn oxidation state [1-3], the concentration of oxygen vacancies grows in the following order: perovskite lattice $\rightarrow$ brownmillerite structure $\rightarrow$ third phase. Migration of oxygen vacancies between the LSMO film and Nb-doped STO substrate was also inspected by STEM-EELS. Supplementary Figures 3c and 3d present STEM-EELS spectra of the O K edge and Ti $L_{2,3}$ edge of the STO substrate. The spectra were recorded straight under the metal tip and 5 nm below the STO/LSMO interface. Three measurements are shown; one for the initial state (i.e. before the application of any voltage pulses), one after formation of the brownmillerite structure in the LSMO film (positive voltage pulse), and one after stabilization of the third LSMO phase (larger positive voltage pulse). The data indicate that the O K edge fine structure damps out and the Ti $L_{2,3}$ edge shifts to lower energy loss after the application of positive voltage pulses and the formation of oxygen-deficient structural phases in the LSMO film. Both effects signify an increase of the oxygen vacancy concentration in STO [4]. From these measurements, we conclude that oxygen vacancies are created in LSMO and STO during the first switching events. After this initial stage, back-and-forth switching between low- and high resistance states weakly modulates the oxygen vacancy concentration in the STO substrate, as illustrated by the STEM-EELS spectra of Supplementary Figures 3e and 3f. In STO, the O K edge fine structure is slightly more pronounced and the Ti $L_{2,3}$ edge appears at higher energy loss when the LSMO film has the brownmillerite structure. Thus, a high concentration of oxygen vacancies in LSMO (brownmillerite phase) corresponds to a reduced concentration of oxygen vacancies in STO and vice versa. To quantify this effect, we compare our data to EELS measurements on oxygen deficient $SrTiO_{3-\delta}$ films [4]. In our spectra, the shift of the Ti $L_{2,3}$ edge amounts to about 0.3 eV. Based on the spectra in Figure 1 of Ref. 4,

this would imply a change in oxygen content of $\Delta\delta \approx 0.1$. This relatively weak effect illustrates that the structural and resistive phase transitions in LSMO are primarily caused by lateral migration of oxygen vacancies within the LSMO film, as illustrated by the preferred horizontal growth direction of new structural phases (Figures 3b,d) and electro-thermal simulations (Figure 5).

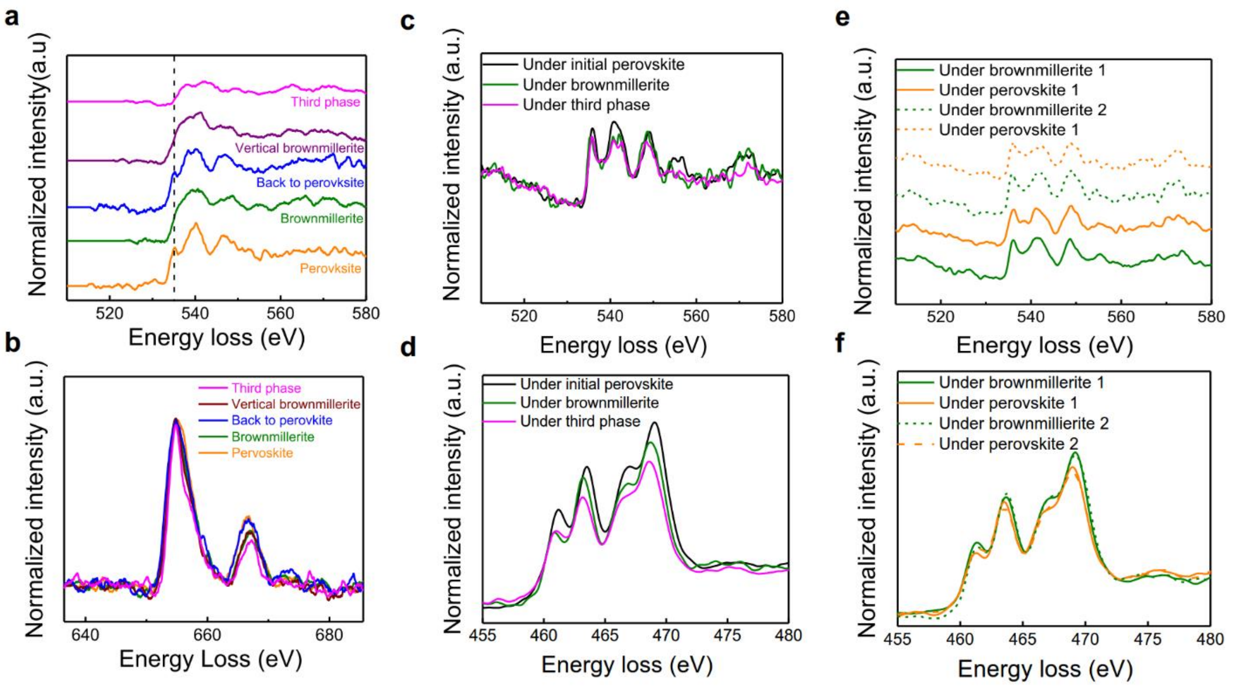

Supplementary Figure 3. a, STEM-EELS spectra of the O K edge for different structural phases of LSMO. The dashed line marks the position of the O K edge pre-peak. **b**, STEM-EELS spectra of the Mn $L_{2,3}$ edge after normalization to the Mn L_3 peak intensity. **c-f**, STEM-EELS spectra of the O-K edge (**c**,**e**) and Ti $L_{2,3}$ edge (**d**,**f**) of the STO substrate (measured 5 nm from the STO/LSMO interface). The spectra on STO are measured during the first switching experiment (2nd column) and during repeated cycling of the voltage pulses (3rd column). For clarity, the curves in (**e**) are plotted with a constant offset.

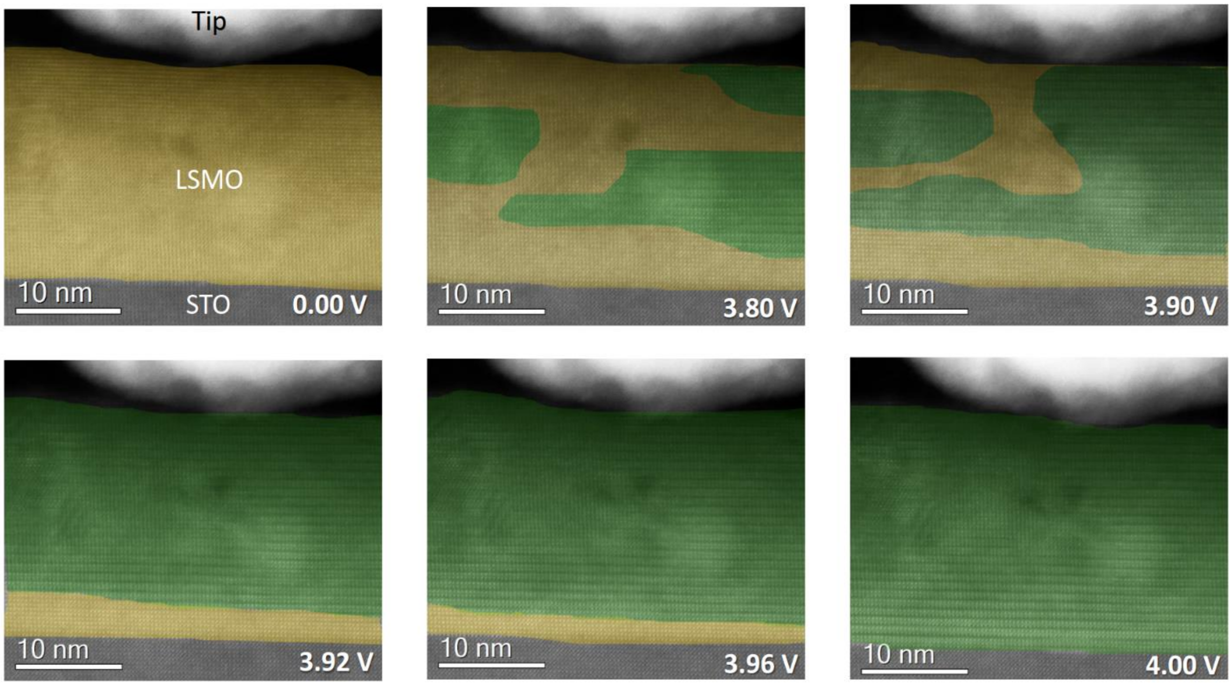

Supplementary Figure 4. STEM images illustrating the transition from the perovskite structure (yellow) to the brownmillerite phase (green) during the first switching event. Oxygen-deficient brownmillerite domains form randomly inside the LSMO film and at the LSMO surface and grow preferentially along the horizontal direction.

Supplementary Note 2. Evolution of lattice spacing in LSMO during resistive switching

High-resolution STEM images recorded during restive switching provide detailed information on the evolution of the LSMO lattice spacing. Supplementary Figure 5 summarizes data for the two-level switching experiments of Figures 1 and 3. Nucleation of the brownmillerite phase at positive voltage pulses rapidly changes the out-of-plane lattice spacing from c = 3.9 Å in the original perovskite lattice to $c_1 \approx 4.9$ Å and $c_2 \approx 3.4$ Å in the newly formed domains. The superstructure within the brownmillerite domains and the surrounding perovskite lattice are both uniform, and the two phases coexist from nucleation at V_p = +3.3 V until saturation at V_p = +4.8 V (see Figure 3b). The structural homogeneity of the brownmillerite domains suggests an even distribution of oxygen vacancies within these areas. Phase

coexistence with well-defined, out-of-plane lattice parameters is also observed when the perovskite lattice structure is reestablished at negative voltage pulses (Supplementary Figure 5b and Figure 3d).

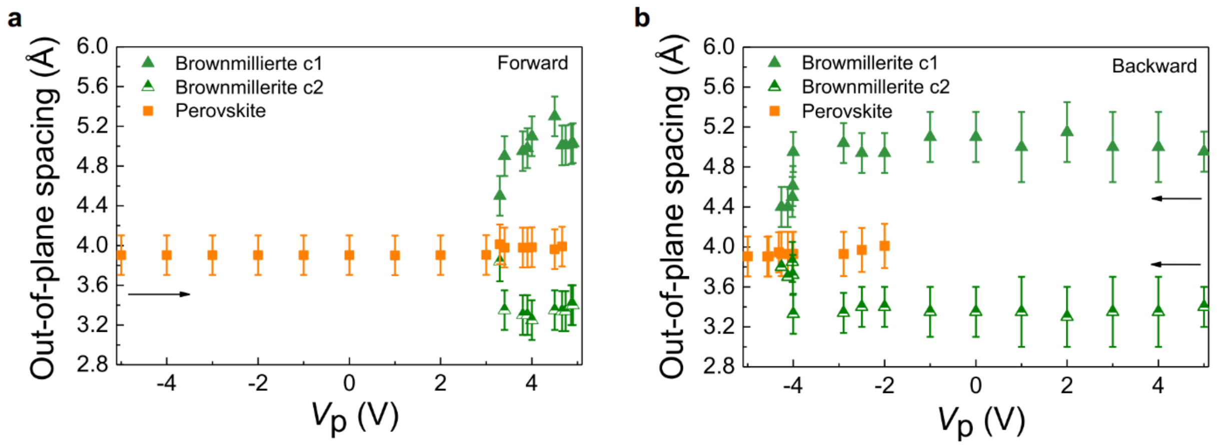

Supplementary Figure 5. a,**b**, Evolution of out-of-plane lattice spacing during the two-level resistive switching experiments of Figures 1 and 3 in the main manuscript. The forward (**a**) and backward (**b**) branches of the switching curves are displayed separately for clarity.

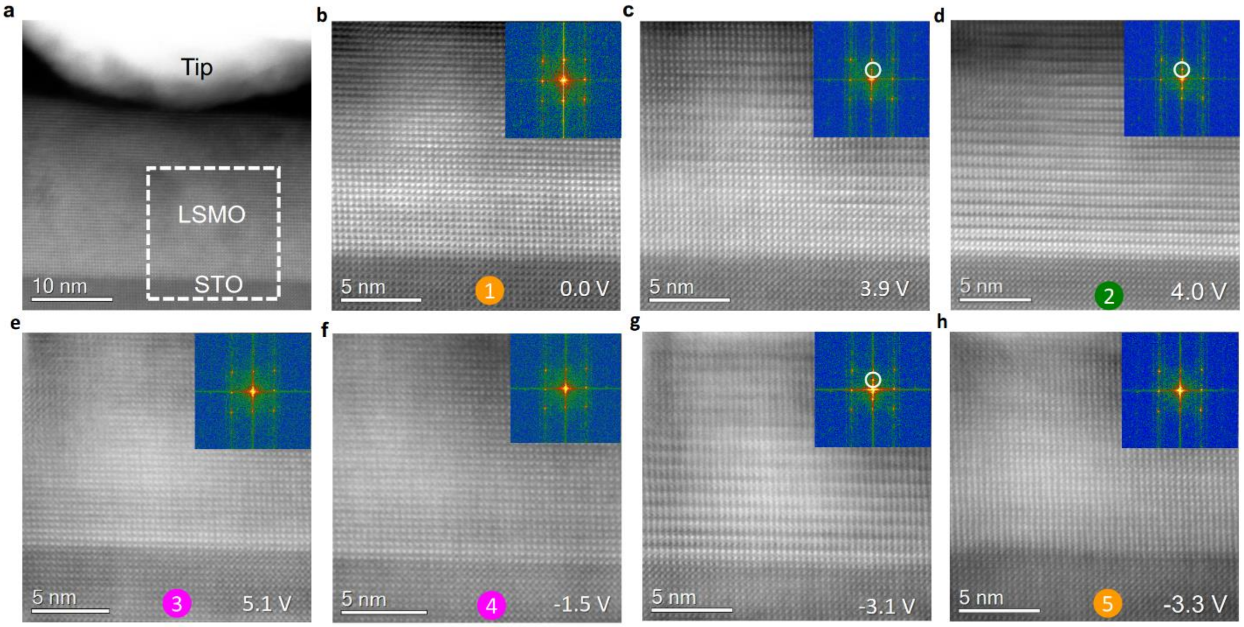

Supplementary Figure 6. STEM images measured during the resistive switching experiments of Figure 4. The colored dots match the indicated resistance states in Figure 4a. **a**, Cross-sectional STEM image of the contact between the metal tip and the LSMO film. **b-h**, STEM-HAADF images and corresponding FFT patterns of the sample within the contact area, as indicated by the dashed line in (**a**), at several stages of the three-level resistive switching process. For negative voltage pulses, a transition from the third phase to the brownmillerite structure occurs only locally and within a narrow voltage range. More details are shown in Supplementary Figure 8.

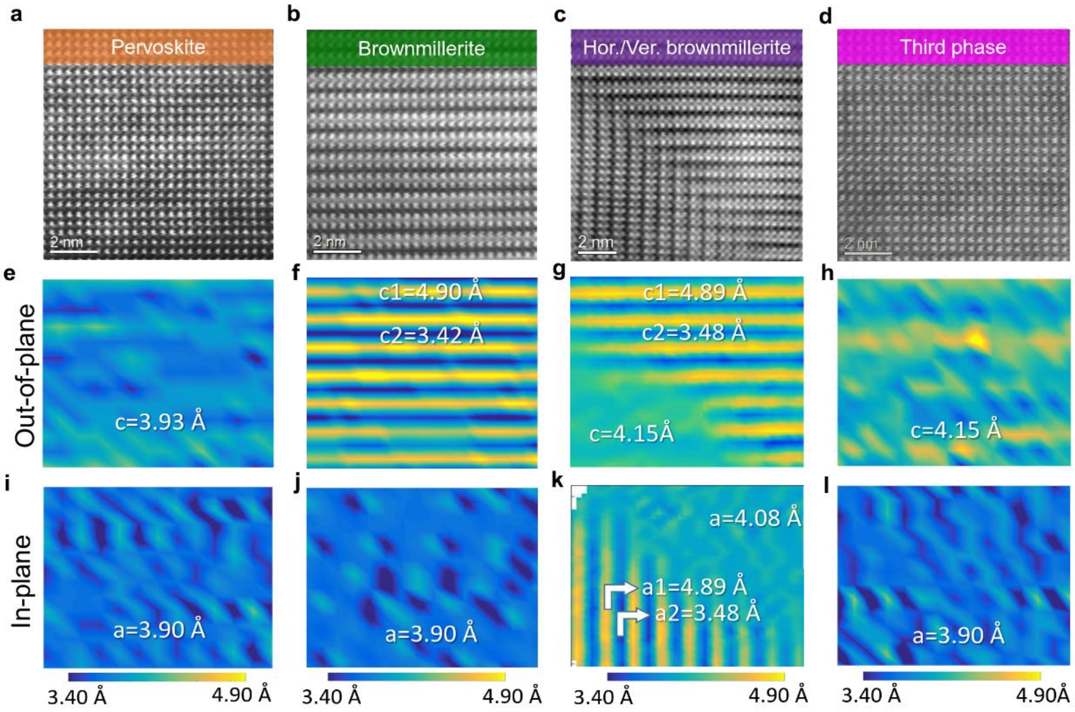

Supplementary Figure 7. High resolution STEM-HAADF images of all structural phases formed during resistive switching in LSMO: **a**, perovskite structure; **b**, horizontal brownmillerite phase; **c**, area with coexisting horizontal and vertical brownmillerite structure; **d**, oxygen deficient perovskite structure with an enhanced out-of-plane lattice spacing of c = 4.15 Å (third phase).

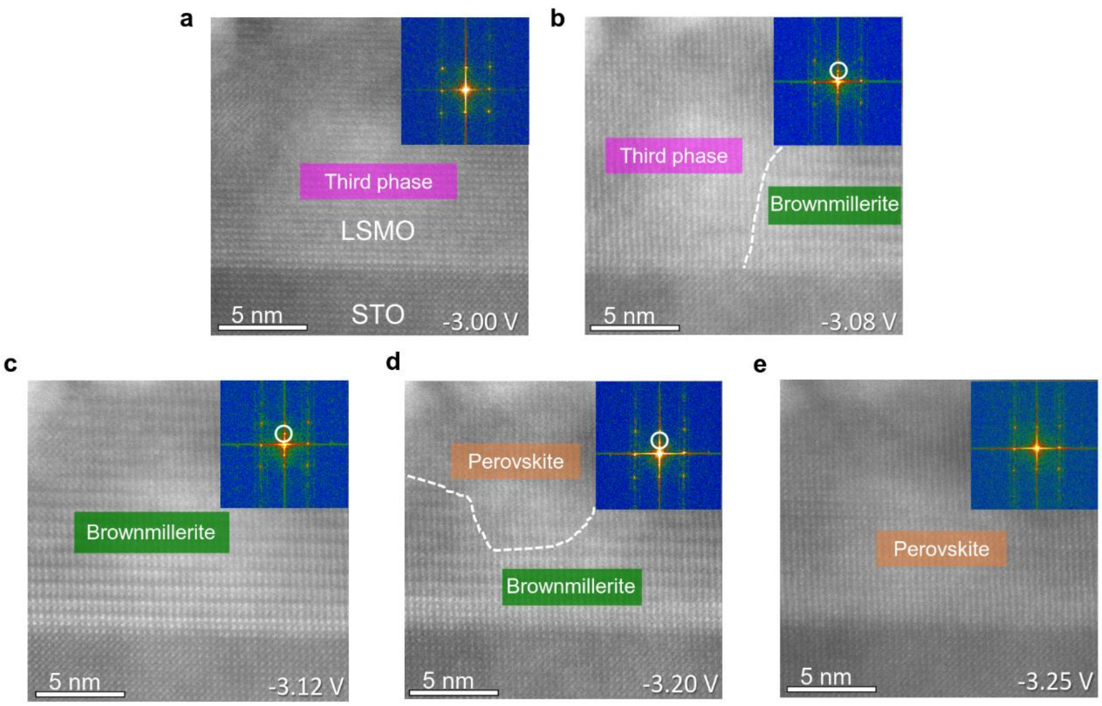

Supplementary Figure 8. a-e, STEM-HAADF images and corresponding FFT patterns of the sample measured during small increments of the negative voltage pulse. The third phase first transforms into the brownmillerite structure and subsequently changes to the original perovskite phase. The entire structural transition occurs within a voltage range of only ~0.2 V. The boundaries between the different phases are indicated by dashed lines in (**b**) and (**d**).

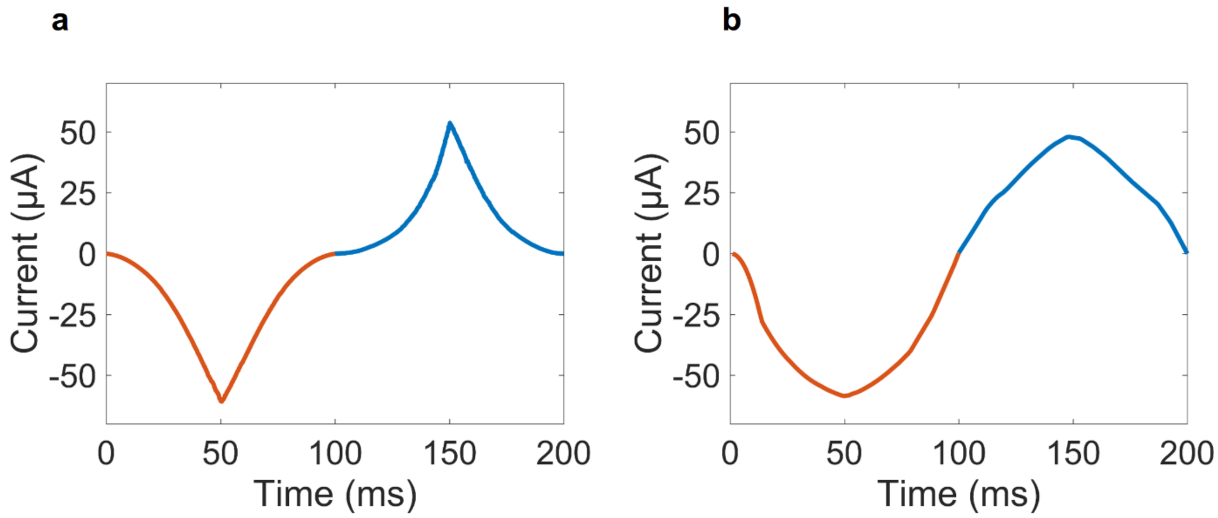

Supplementary Figure 9. a, Experimentally measured current pulse during the application of a 100 ms triangular voltage pulse with V_p = –4.0 V (red) and V_p = +4.4 V (blue). The data correspond to the switching experiments of Figure 1 in the main manuscript. **b**, Simulated current pulse during the application of a triangular voltage pulse in the thermo-electric simulations of Figure 5. The simulated maximum current closely corresponds to the experimental data in (**a**).

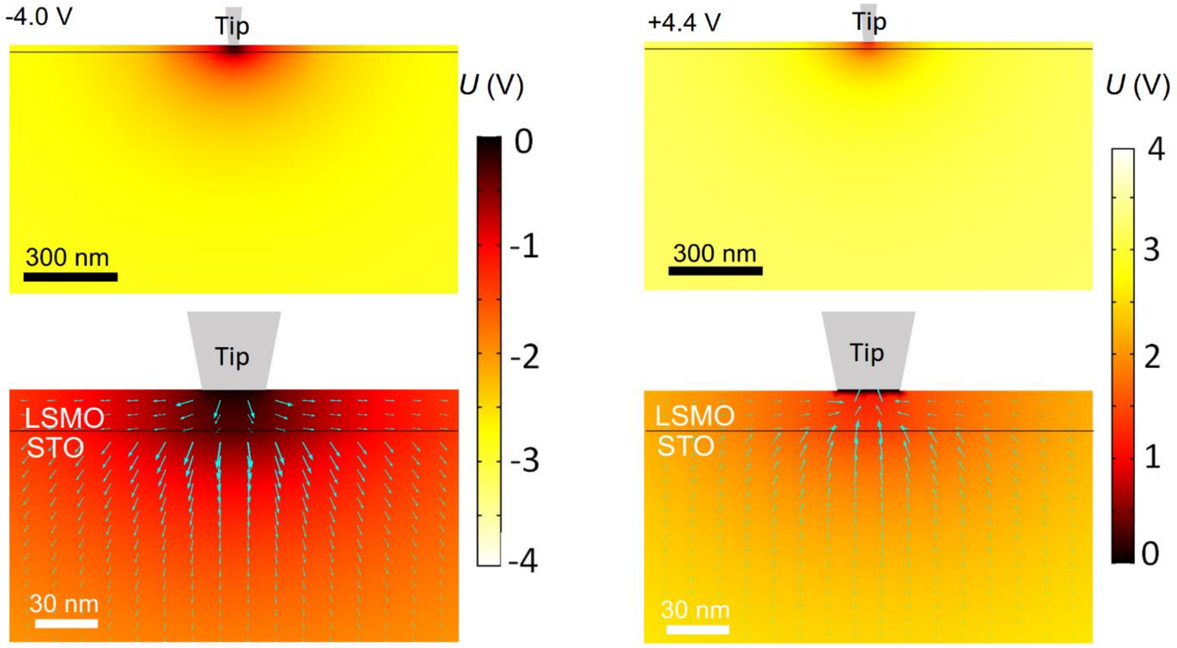

Supplementary Figure 10. Simulated electric field and potential maps. All color maps show the electric potential and the arrows in the lower panels indicate the direction and magnitude of electric field. The maps are recorded when negative (left) and positive (right) triangular voltage pulses reach their maximum value.

Supplementary Note 3. Stability of structural phases and resistance states

The stability of the different structural phases after resistive switching was monitored using *in situ* TEM. Supplementary Figure 11 shows an example for the brownmillerite phase. In this experiment, the original perovskite phase is first transformed into the brownmillerite structure by a positive voltage pulse of V_p = +4.5 V. As a result, the resistance increases. The resistance and lattice structure are subsequently monitored at a small measurement voltage of V_m = 0.2 V. The resistance remains constant for 155 minutes (Supplementary Figure 11a), after which it increases due to an abrupt disconnect between the metal tip and the LSMO film (see Supplementary Figure 11d). The change in resistance is thus not caused by an instability of the brownmillerite phase but, rather, by an artefact in the *in situ* TEM measurement. The

brownmillerite structure remains stable well beyond the duration of this experiment. In a separate test, a TEM specimen with brownmillerite LSMO film was stored for two months. STEM-HAADF imaging after two months did not reveal any relaxation into the original perovskite phase (Supplementary Figure 12). The brownmillerite structure and accompanying high resistance state that are induced by combined effects of applied electric field and Joule heating are thus stable over time. The stability of the third phase was also assessed. Supplementary Figure 13 summarizes an experiment wherein the perovskite LSMO film is first transformed into a coexisting brownmillerite and third phase. STEM imaging of the same area after 10 days (Supplementary Figure 13d) reveals that the boundary between the two structural phases has only meandered a bit in favor of the brownmillerite structure. This observation suggests very slow re-ordering of oxygen vacancies near the phase boundary.

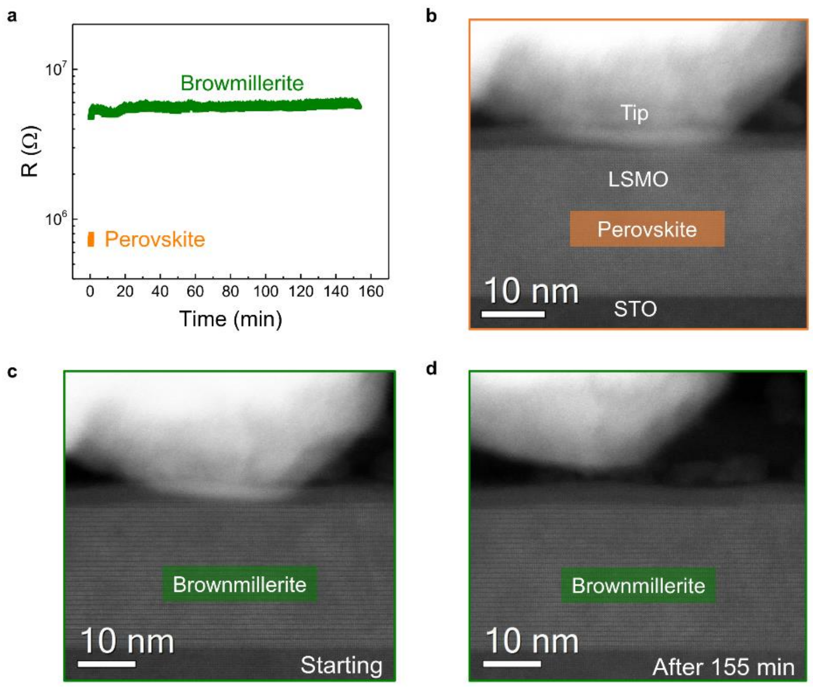

Supplementary Figure 11. a, Resistance versus time for a LSMO film with a brownmillerite structure. In this experiment, the brownmillerite phase is first induced by a positive voltage pulse of V_p = +4.5 V and the resistance is subsequently monitored at a measurment voltage of V_m = 0.2 V. **b-d**, STEM images of the metal tip/LSMO contact area during several stages of the experiment.

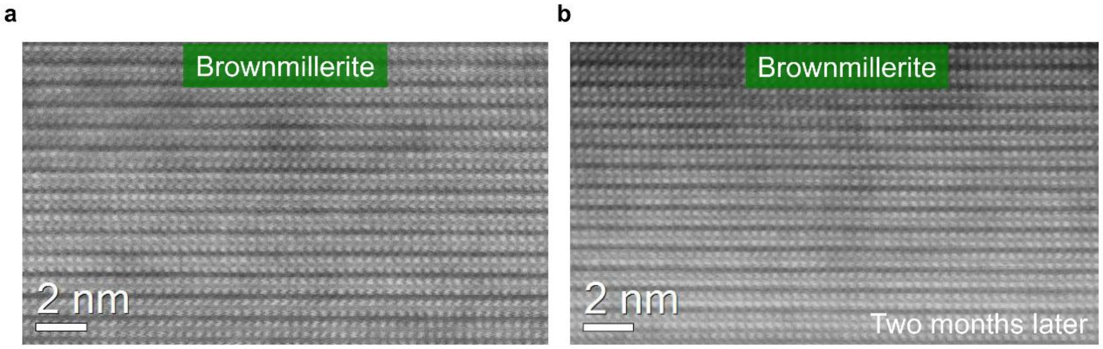

Supplementary Figure 12. **a**,**b**, STEM-HAADF images of a LSMO film immediately after switching to the brownmillerite phase (**a**) and after storing the sample for two months in a vacuum desiccator (**b**).

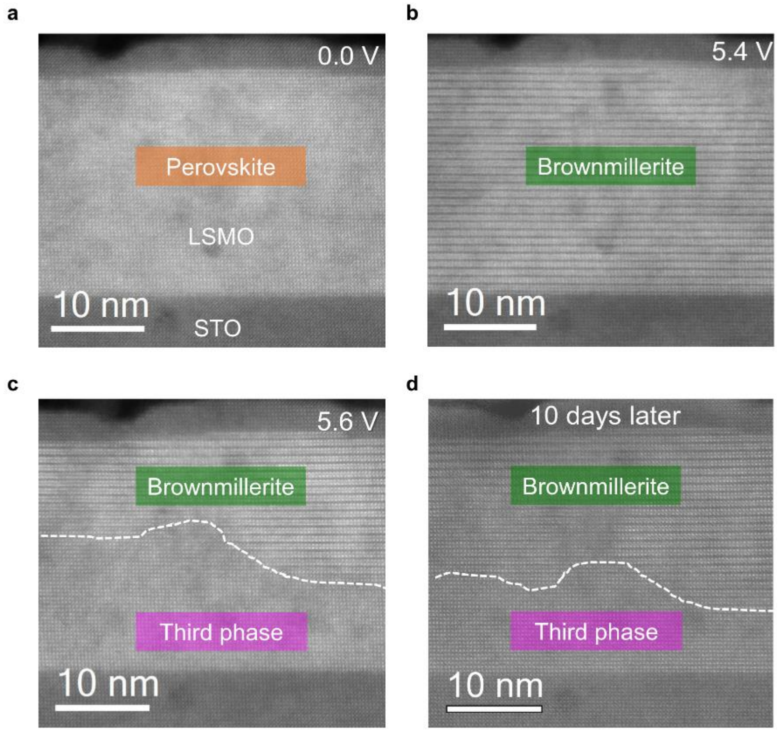

Supplementary Figure 13. **a-c**, STEM images measured during a three-level resistive switching experiment. **d**, STEM image of the same area after 10 days. The boundary between the brownmillerite and third phase is indicated by a dashed line in (c) and (d).

Supplementary References

[1] Varela, M. *et al*. Atomic-resolution imaging of oxidation states in manganites. *Phys. Rev. B* **79**, 085117 (2009).

[2] Li, Z. *et al*. Interface and surface cation stoichiometry modified by oxygen vacancies in epitaxial manganite films. *Adv. Funct. Mater*. **22**, 4312-4321 (2012).

[3] Yao, L. *et al*. Electron-beam-induced perovskite-brownmillerite-perovskite structural phase transitions in epitaxial $La_{2/3}Sr_{1/3}MnO_3$. *Adv. Mater.* **26**, 2789-2793 (2014).

[4] Muller, D.A, *et al.* Atomic-scale imaging of nanoengineered oxygen vacancy of profiles in $SrTiO_3$, *Nature*, **430**, 657-661 (2004).
